# Effects of small interfering RNAs targeting fascin on human esophageal squamous cell carcinoma cell lines

**DOI:** 10.1186/1746-1596-5-41

**Published:** 2010-06-21

**Authors:** Cristian M Ortiz, Tetsuo Ito, Yosuke Hashimoto, Satoshi Nagayama, Akira Iwai, Shigeru Tsunoda, Fumiaki Sato, Miguel Martorell, Jose Angel Garcia, Ana Perez, Yutaka Shimada

**Affiliations:** 1 Department of Pathology, Valencia University, Hospital General Universitario de Valencia, Avenida tres cruces N° 2, CP 46014, Valencia, Spain; 2 Department of of Surgery, Sugita Genpaku Memorial Obama Municipal Hospital. 2-2 Ohte-cho, Obama, Fukui, 917-8567, Japan; 3 Division of Colon and Rectal Surgery, Shizuoka Cancer Center Hospital, 1007 Shimonagakubo, Nagaizumi-cho, Sunto-gun, Shizuoka 411-8777, Japan; 4 Department of Surgery, Graduate School of Medicine, Kyoto University, Kawaracho 54 Shogoin, Sakyo-ku, Kyoto, 606-8507, Japan; 5 Department of Nanobio Drug Discovery, Graduate School of Pharmaceutical Sciences, Kyoto University, Kawaracho 54 Shogoin, Sakyo-ku, Kyoto, 606-8507, Japan; 6 Department of Surgery & Science. Graduate School of Medicine & Pharmaceutical Sciences for Research, University of Toyama, 2630 Sugitani, Toyama, 930-0194, Japan

## Abstract

**Background:**

Fascin induces membrane protrusions and cell motility. Fascin overexpression was associated with poor prognosis, and its downregulation reduces cell motility and invasiveness in esophageal squamous cell carcinoma (ESCC). Using a stable knockdown cell line, we revealed the effect of fascin on cell growth, cell adhesion and tumor formation.

**Methods:**

We examined whether fascin is a potential target in ESCC using *in vitro *and *in vivo *studies utilizing a specific siRNA. We established a stable transfectant with downregulated fascin from KYSE170 cell line.

**Results:**

The fascin downregulated cell lines showed a slower growth pattern by 40.3% (p < 0.01) and detachment from collagen-coated plates by 53.6% (p < 0.01), compared to mock cells, suggesting that fascin plays a role in cell growth by maintaining cell adhesion to the extracellular matrix. *In vivo*, the tumor size was significantly smaller in the tumor with fascin knockdown cells than in mock cells by 95% at 30 days after inoculation.

**Conclusions:**

These findings suggest that fascin overexpression plays a role in tumor growth and progression in ESCC and that cell death caused by its downregulation might be induced by cell adhesion loss. This indicates that targeting fascin pathway could be a novel therapeutic strategy for the human ESCC.

## Background

Esophageal squamous cell carcinoma (ESCC) is one of the most prevalent cancers worldwide [1,2]. One of the reasons for its poor prognosis is that ESCC is difficult to diagnose at an early stage and exhibits local invasion and lymph node metastasis in its advanced stage, even at initial diagnosis [3]. The capability of tumor invasion and metastasis is the result of several sequential steps including motile properties of tumor cells to overcome cell-cell and cell-matrix adhesion and to invade the surrounding tissue [4-6].

Invasive tumor cells often show morphological changes, such as membrane protrusions as well as the loss of cell-cell adhesion and the loss of junctional communications, which probably result from rearrangements of the cytoskeletal microfilaments by the action of actin cross-linking proteins [7-9]. Fascin is a highly conserved 55-kDa actin bundling protein that plays an important role in the organization of several types of actin-based structures such as filopodia, lamellipodial ribs, dendrites, spikes and microvilli [10]. It was originally found in extracts of unfertilized sea urchin eggs and localized to microfilament bundles within microvilli cores and within filopodia on the surface of fertilized sea urchin eggs [11]. Fascin is normally expressed in cells inducing membrane protrusions and increased cell motility, such as neurons, glial cells and dendritic cells [12-14] or migrating cells such as endothelial cells and macrophages [14]. The fascin expression level in epithelial cells is usually low, however it is often upregulated and overexpressed in transformed cells and in several types of human neoplasm, including breast [15,16], ovary [17], skin [18], pancreas and liver [19-21], non-small cell lung carcinomas, lung carcinoids [22,23], stomach and esophageal squamous cell carcinoma [18,24,25]. We have previously reported that actin bundling protein fascin and its overexpression was related to poor prognosis, as well as that its stable knockdown reduces cell motility and invasiveness in the ESCC [26]. We have also reported that fascin immunoreactivity in gastric carcinoma was associated with lymph node metastasis [27]. In order to determine whether fascin is a potential gene target, we performed *in vitro *and *in vivo *studies. In the present study, using a stable knockdown cell line, we elucidated the role of fascin in cell growth and cell adhesion *in vitro *and revealed the effect of fascin knockdown on the tumor growth in the *in vivo *tumor formation assay. These results imply a potentially important role of fascin inhibition in the treatment of ESCC and the prevention of tumor invasion and metastasis.

## Methods

### Cell culturing

All human esophageal squamous carcinoma cell lines KYSE 170 parental (wild) and derivate cells (KYSE 170mock, KYSE 170F3 and KYSE 170F4) were established in our laboratory and maintained in RPMI 1640 (Life Technologies, Gaithersburg, MD) and Ham's F12 (Nissui Pharmaceutical, Tokyo, Japan) with 2% fetal bovine serum (FBS).

### Western blot analysis

Whole-cell extract lysate was prepared from 1 × 10^7 ^cells in a sample buffer (2% sodium dodecyl sulfate [SDS], 10% glycerol, 50 mM Tris-Hcl, pH 6.8) at room temperature. Cell lysates were sonicated and the protein content was measured with the Bradford method using BCA Protein Assay Reagent (Pierce, Rockford, MA), after which the cell lysates were electrophoresed on a 12% polyacrylamide gel SDS page and transferred to a polyvinylidene difluoride membrane (Immobilon, Milipore, Bedford, MA) using a semidry transfer blot system (Bio-Rad, Hercules, CA). The membranes were blocked with TBS (20 mM Tris, 150 mM NaCl, pH 7.6) containing 1% Tween 20 and 5% skimmed milk for 1 hour. The membranes were incubated overnight at 4°C with anti-human fascin mouse monoclonal antibody (DAKO, Osaka, Japan; diluted 1:500) or with anti-human β-actin mouse monoclonal antibody (Sigma Inc., St. Louis, MO; diluted 1:2000) as an internal control. They were washed and then incubated at room temperature for 1 hour with anti-mouse IgG-HRP (Zymed, San Francisco, CA), as a secondary antibody, and analyzed using ECL plus reagent (Amersham, Buckinghamshire, UK).

### Purification of total cellular mRNA and Reverse Transcription-PCR

Total RNA was extracted from KYSE cell lines by the TRIzol reagent (Invitrogen, Carlsbad, CA) according to the manufacturer's protocols [28,29]. Reverse transcription of total cellular RNA was performed using a First-Strand cDNA Synthesis Kit (Amersham, Buckinghamshire, UK). cDNA was subjected to PCR for 25 cycles of amplification using an Advantage cDNA PCR kit (Becton Dickinson Biosciences, Palo Alto, CA). Each PCR cycle consisted of a denaturation step for 1 minute at 94°C and an annealing step for 1 minute at 60°C. The final extension step was carried out for 5 minutes at 72°C. The PCR products were separated on 1.5% agarose gel and visualized by ethidium bromide staining. PCR primers used for fascin were 5'- AGGCGGCCAACGAGAGGAAC-3' as the forward primer and 5'-ACGATGATGGGGCGGTTGAT-3' as the reverse primer; and for glyceraldehydes-3-phosphate dehydrogenase (G3PDH), 5'-TGGTATCGTGGAAGGACTCATGAC-3' was used as the forward primer and 5'-ATGCCAGTGAGCTTCCCGTTCAGC-3' as the corresponding reverse primer. cDNA from HeLa cells was used as a positive control for each analysis.

### Cell growth assay

Cells were plated into 6 cm dishes (2 × 10^4 ^cells per dish) at day 0 and incubated for 24 hours for sufficient cell growth. Cells were harvested with trypsin/EDTA every 48 hours for five days, and counted with a cell counter (Coulter Z1, Beckman Coulter, Fullerton, CA). To examine the effect of the suppression of fascin expression on cell growth, we compared it with the control culture in triplicate. Each experiment was repeated three times independently.

### Adhesion assay

Cells were seeded into collagen-coated plates (Becton Dickinson, MA) without FBS. After 24 hours of incubation, the adherent and floating cells were counted from five randomly selected fields. The assay was repeated three times under the same conditions.

### Caspase inhibition

200 μmol/L Z-VAD-FMK (BD Biosciences, San Jose, CA) was added to the cells after the cells were plated. The inhibition of cell growth was measured by MTT assay and cell counting assay. The assay was repeated three times under the same conditions.

### Construction of fascin-small interfering RNA vector and transfection

In order to construct a vector for fascin-small interfering RNA (siRNA), pSilencer2.1-U6 hygro (Ambion, Inc., Austin, TX) was digested with *Bgl*II and *Hin*dIII. A chemically synthesized oligonucleotide encoding a fascin-short hairpin siRNA that included a loop motif was inserted downstream of the U6 promoter of the plasmid using a DNA ligation kit (Takara Bio, Inc., Shiga, Japan), and was cloned. Sequences of the oligonucleotide targeted to fascin were 5'-GCCUGAAGAAGAAGCAGAU-3' corresponding to positions 116 to 134 within fascin exon 1. An ESCC cell line KYSE170 was stably transfected with either the fascin-siRNA expression vector or the negative control vector (pSilencer2.1-U6 hygro) using FuGene6 reagent (Roche Diagnostics, Basel, Switzerland) [26], and cell clones were selected against 100 μg/mL hygromycin (Nacalai Tesque, Kyoto, Japan).

### Tumor formation assay in nude mice

Suspensions of 1 × 10^6 ^KYSE 170 parental (wild) and derivate cells (KYSE 170mock, KYSE 170F3 and KYSE 170F4) in PBS (60 μL) were injected subcutaneously into the right flanks of 5-week-old male BALB/c nu/nu mice (Japan SLC, Shizuoka, Japan) at day 0. The inoculation was conducted in five mice per group, and tumor growth was estimated from the average volume of tumors by the formula: 1/2 × L^2 ^× W (where L is the length of the tumor and W is the width of the tumor). At 30 days after injection, all mice were sacrificed and the subcutaneous tumors were resected and fixed in 10% formaldehyde/PBS. Tumors were then embedded in paraffin and stained with H&E and fascin. All animal experiments were performed in accordance with institutional guidelines.

### Immunohistochemical staining

Resected tumors from the *in vivo *experiments were fixed in 10% formaldehyde solution, embedded in paraffin, cut in 4 μm thick sections, and mounted on aminopropyltriethoxylane-coated glass slides. Immunohistochemical staining was carried out using an Envision Kit (Dako Cytomation, Glostrup, Denmark). Tissue sections were incubated overnight at 4°C with anti-human fascin monoclonal antibody clone 55kDa (DAKO, Osaka, Japan; 1:50 dilution) and then incubated with biotinylated anti-mouse IgG for 30 minutes at room temperature. Tissue sections were then stained with 3,3' diaminobenzidine liquid system (Dako Cymation, Glostrup, Denmark), counterstained with Mayer's haematoxilyn, dehydrated and mounted. We performed the same protocol using goat anti-human caspase 3 polyclonal antibody (Santa Cruz Biotechnology, CA, USA; dilution 1:500). As a negative control, the primary antibody was replaced with normal mouse IgG, and a further control was carried out without the primary antibody.

### TUNEL assay

The DeadEnd Colorimetric TUNEL system kit was used for the TUNEL assay. The paraffin-embedded tissues were fixed in 4% formaldehyde with PBS for 15 minutes, permeabilized with 20 μg/ml proteinase K for 15 minutes and subsequently incubated at 37°C for 60 minutes with the rTdT reaction mix on the slide. Streptavidin HRP solution 1:500 in PBS was added and incubated for 30 minutes. DAB solution was then added for 5 minutes. Then the apoptotic cells were counted in five different fields at 40 ×, obtaining the average and standard deviation (SD).

### Statistical analysis

The statistical analysis was performed using the software StatView 4.5 (Abacus Concept, Berkley, CA). A p-value of < 0.05 indicated statistical significance.

## Results

### Downregulation of fascin expression by siRNA in ESCC cell lines

**Figure 1:**
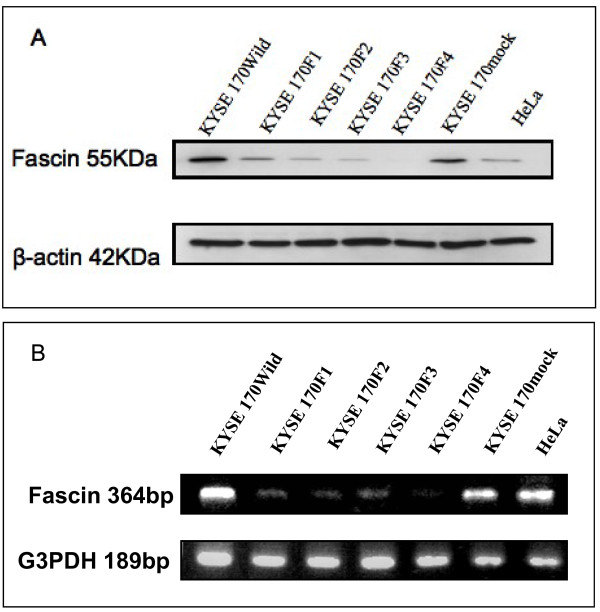
**Expression of fascin in parental KYSE cells and stable transfectant with siRNA. **(A) Western blot analysis in parental KYSE 170 cells and stable transfectant with siRNA. The HeLa cell line was included as a positive control for fascin expression. (B) Semiquantitative reverse transcription-PCR analysis for fascin expression in parental KYSE 170 cells and stable transfectant with siRNA. The HeLa cell line was included as a positive control in the same analysis.

The expression of fascin was reduced by 74.8% in the stable subclones 170F1, 89.1% in 170F2, 93% in 170F3 and 97.7% in 170F4. The transfection for the nonspecific siRNA vector (170 mock) did not affect the expression of fascin. HeLa cells were used as a positive control for fascin (Figure 1A). Semiquantitative reverse transcription-PCR was performed, obtaining similar results to western blot analysis (Figure 1B).

### siRNA against fascin decreased cell growth of ESCC *in vitro*

**Figure 2:**
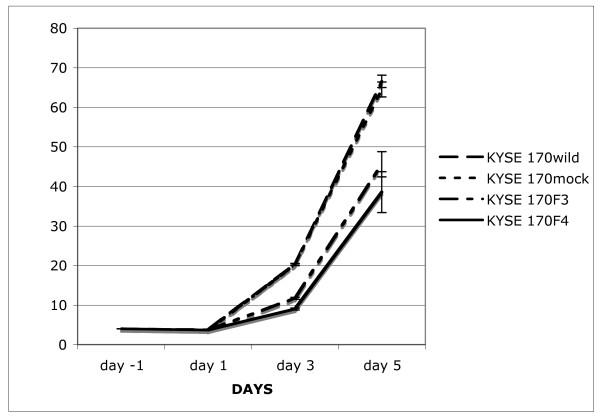
**Vector based siRNA for fascin knockdown in the ESCC cell line**. *In vitro *growth assay in KYSE 170 cells and stable transfectant cells with an empty vector (KYSE 170 mock) or fascin-siRNA vector (KYSE 170F3 and KYSE 170F4). The cells were counted every two days and the experiment was performed three times.

We hypothesized that knockdown of fascin might affect cell growth. In order to verify this hypothesis, we investigated the effect of fascin knockdown on proliferation in one ESCC cell line (KYSE 170). With the suppression of fascin, the cell growth was inhibited by 40.3% in KYSE 70F4 at the day 5, as compared to the empty vector siRNA transfected cell line (KYSE 170 mock) (p < 0.01) (Figure 2).

### siRNA against fascin decreased cell adhesion of ESCC *in vitro*

**Figure 3:**
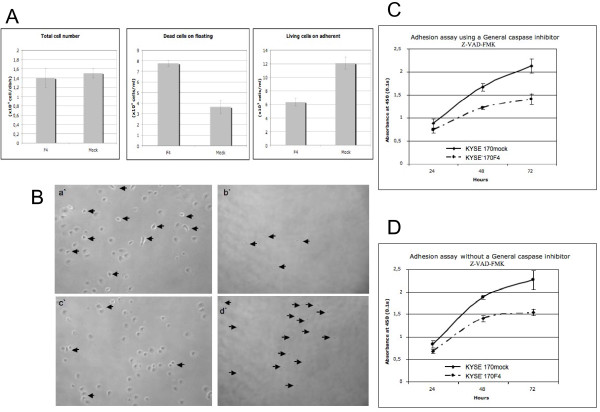
**Adhesion assay of  stable transfectant cells with an empty vector (KYSE 170 mock) compared to a siRNA vector against fascin (KYSE 170F4). **(A) Total number of cells, number of floating dead cells and number of adherent living cells for stable transfectant cells with an empty vector (KYSE 170 mock) as compared to a siRNA vector against fascin (KYSE 170F4). Left, the total number of cells (× 10^4 ^cells/dish). Middle, the number of floating dead cells (× 10^3 ^cells/ml). Right, the number of adherent living cells (× 10^3 ^cells/ml). (B) Photographs of ESCC 24 hours after seeding. (a') Adherent stable transfectant cells with an empty vector (KYSE 170 mock). (b') Floating stable transfectant cells with an empty vector (KYSE 170 mock). (c') Adherent stable transfectant cells with a siRNA vector against fascin (KYSE 170F4). (d') Floating stable transfectant cells with a siRNA vector against fascin (KYSE 170F4). (C) Effect of fascin knockdown and the effect of caspase dependency in cell growth. The cells were treated with 200 μmol/L of the general caspase inhibitor Z-VAD-FMK (+). (D) Control, without the general caspase inhibitor Z-VAD-FMK (-).

In the cell adhesion *in vitro *experiment, the differences between KYSE 170 mock and fascin knockdown cells were not observed until 6 hours after seeding. The KYSE 170 mock cells attached well after 6 hours, whereas the fascin knockdown cells (KYSE 170F4) did not attach properly and an increasing number of cells was floating. After 24 hours of seeding, the number of adherent living cells was significantly lower in the KYSE 170F4 group than in the KYSE 170 mock group (Figure 3A); in contrast, the number of floating cells increased (Figure 3B). As a result, the total number of cells decreased in the fascin knockdown group. These results suggest that fascin knockdown induces the loss of cell adhesion to the matrix and might result in the inhibition of cell growth in ESCC cells.

### Adhesion assay using a general caspase inhibitor (Z-VAD-FMK)

The general caspase inhibitor (Z-VAD-FMK) did not affect the inhibition of attachment induced by fascin knockdown (Figures 3C and 3D). The difference in cell count at 72 hours between the fascin knockdown cell line and the mock cell line remained the same, both using the general caspase inhibitor and without (mean 40.1% both). These results provide convincing evidence that detachment may be caused by fascin knockdown.

### Effect of fascin on pFAK and Integrin α2 expression *in vitro*

**Figure 4:**
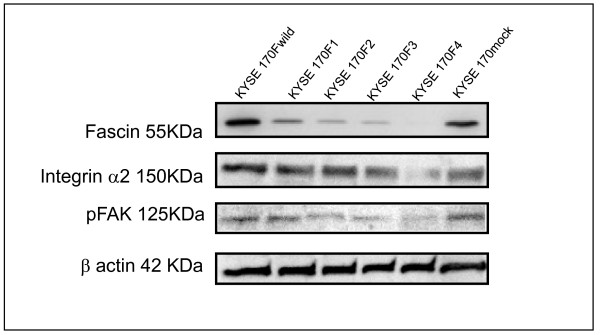
**Western blot analysis for the expression of fascin, pFAK and integrin α2 proteins in parental KYSE170 cells and the stable transfectant with siRNA**.

Using the in vitro model and western blot analysis, we observed a decreasing expression of the two proteins involved in attachment and cell cycle signaling pFAK and Integrin α2 in all the studied clones, with the highest expression in KYSE 170wild and mock, and the lowest expression in KYSE 170F4 cells (Figure 4). These results suggest that the occurrence of interactions between fascin and other proteins or membrane proteins may occur.

### Effect of fascin knockdown on tumor formation *in vivo*

**Figure 5:**
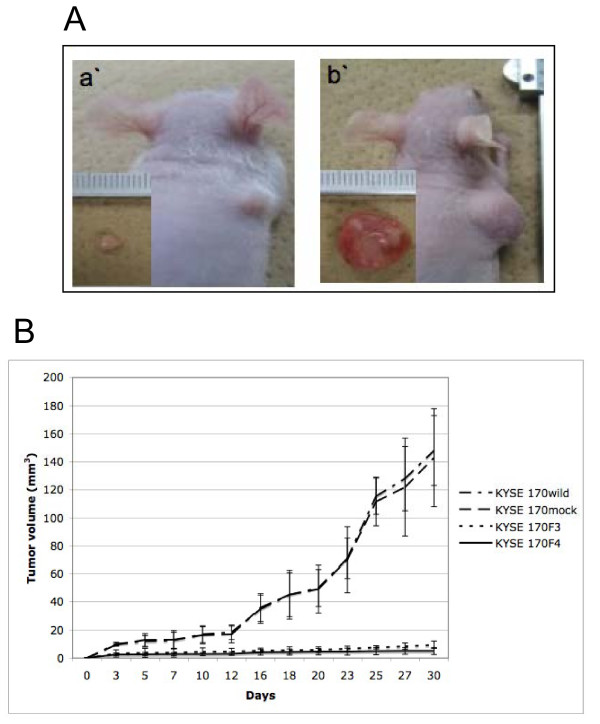
**The effect of fascin knockdown on tumor formation model**. (A) Tumor subcutaneous implantation 30 days after inoculation. (a') Fascin knockdown cells (KYSE 170F4) were injected subcutaneously in the right flank. (b') The KYSE 170 mock cells were injected in the right flank. (B) Tumor growth curves of mice injected with KYSE 170 wild type cells, empty vector-transfected cells (KYSE 170 mock) and fascin siRNA-transfected cells (KYSE 170F3 and KYSE 170F4).

**Figure 6:**
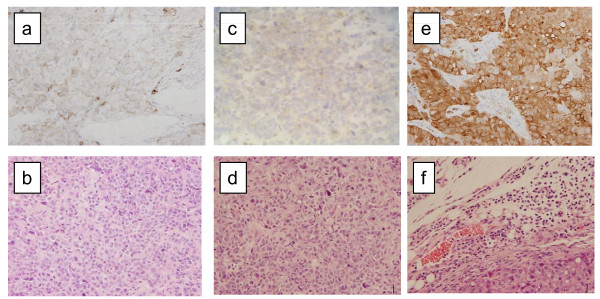
**Immunohistochemical expression of fascin in subcutaneous tumors. **(a) Fascin immunohistochemical staining of subcutaneous tumors 30 days after inoculation of fascin siRNA transfected cells (KYSE 170F3).. (b) H&E staining of subcutaneous tumors 30 days after inoculation of fascin siRNA transfected cells (KYSE 170F3). (c) Fascin immunohistochemical staining of subcutaneous tumors 30 days after inoculation of fascin siRNA transfected cells (KYSE 170F4). (d) H&E staining of subcutaneous tumors 30 days after inoculation of fascin siRNA transfected cells (KYSE 170F4). (e) Fascin immunohistochemical staining of subcutaneous tumors 30 days after inoculation of the empty vector-transfected cells (KYSE 170 mock). (f) H&E staining of subcutaneous tumors 30 days after inoculation of the empty vector-transfected cells (KYSE 170 mock). The assay was performed using five mice per group.

In order to investigate the possible effect of fascin-siRNA on tumor formation *in vivo*, we performed a subcutaneous tumor formation assay in nude mice (Figure 5A). We evaluated tumor volume and weight at 30 days after inoculation. The tumor formations caused by fascin-siRNA transfected cells (KYSE 170F3 and KYSE 170F4) were evidently smaller than those formed by KYSE 170wild and KYSE 170 mock (Figure 5B). The average tumor volume was significantly lower in KYSE 170F3 and KYSE 170F4 by 91% and 95%, respectively, compared to KYSE 170 mock (p < 0,0001 and p < 0,0001, respectively). The average tumor weight was also inhibited in KYSE 170F3 and KYSE 170F4 by 87% and 90%, respectively (p < 0,0004 and p < 0,0003, respectively). Finally, all tumors were stained with H&E and fascin, and fascin protein expression was reduced to a minimum in tumors of inoculated mice with fascin-siRNA transfected cells, although not in tumors of empty vector transfected cells (KYSE 170 mock) (Figure 6).

### siRNA for fascin induced apoptosis of ESCC *in vivo*

**Figure 7:**
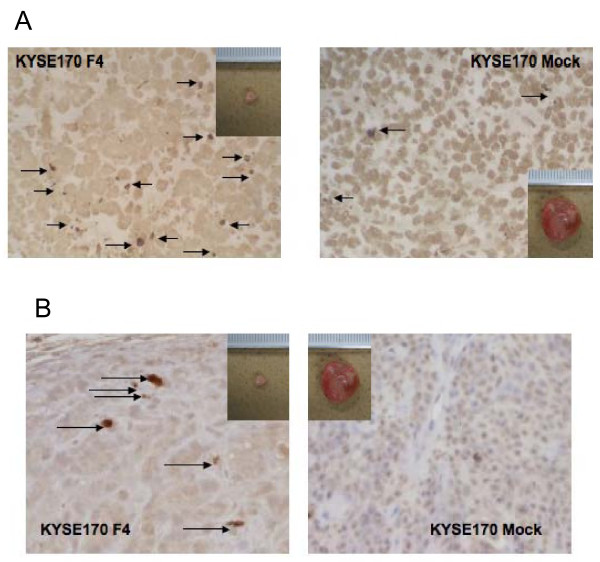
**Detection of apoptotic cells in subcutaneous tumors by TUNEL assay and immunohistochemical staining for caspase 3. **(A) TUNEL assay detecting apoptotic cells (arrows) using stable transfectant cells with an empty vector (KYSE 170 mock) and with a siRNA vector against fascin (KYSE 170F4). (B) Photomicrographs of immunohistochemical staining detected active caspase 3 pattern (arrows) in the *in vivo *model tumor using stable transfectant cells with an empty vector (KYSE 170 mock) and with a siRNA vector against fascin (KYSE 170F4).

The analysis of tumoral apoptosis was conducted by TUNEL assay (Figure 7A) and immunohistochemistry for caspase 3 (Figure 7B) where the number of apoptotic cells was higher in the fascin downregulated tumor than in the KYSE 170 mock tumor. For the TUNEL assay, the average of apoptotic cells in the fascin knockdown tumor was 15 ± 3.6 cells per high power field and for the KYSE 170 mock tumor it was 2 ± 0.83 cells per high power field.

## Discussion

Although there are many reports demonstrating the involvement of fascin in carcinogenesis, its biological role and the relationship with the different oncogenic pathways in tumorigenesis is still unclear. In this study, we showed that fascin knockdown strongly inhibits cell growth and cell adhesion abilities of the ESCC cell line KYSE 170 *in vitro *and *in vivo*. This is most likely due to focal detachment to the matrix of tumoral cells, suggesting that the occurrence of interactions between fascin and other proteins or membrane proteins may occur. The ability of tumor cells to migrate might be connected to their tumorigenicity, and fascin might participate in pathways regulating migration, cell growth and detachment-induced apoptosis (anoikis) [25,30-32].

The changes involved in cell proliferation might be crucial in regulating tumor growth and progression. In our experimental design, *in vitro *studies showed that the inhibition of cell growth by fascin knockdown was time dependent, visible evidence in the cell number was not observed until 24 hours after seeding. Furthermore, the caspase general inhibitor did not affect the inhibition of growth caused by fascin knockdown. These results suggest that the inhibition of cell adhesion caused by fascin knockdown results in the inhibition of cell growth. Fascin knockdown cells strongly inhibited tumor growth *in vivo*. Although this inhibition might be the result of several factors, our data for TUNEL assay and caspase 3 immunohistochemical staining for the *in vivo *experiment confirmed that apoptosis was higher in the fascin knockdown cells. The mechanism by which apoptosis is induced by fascin knockdown is unclear. This data supports our suggestion that detachment might be a cause, rather than a result of apoptosis, resulting in the inhibition of cell growth. However, the results on the *in vitro *growth assay were rather different compared with the *in vivo *growth assay, where the inhibition of the cell growth *in vitro *was lower than in the *in vivo *study. There are several mechanisms involved in the xenograft model, and the tumor growth is strongly associated with the tumor microenvironment, including the extracellular matrix and cellular components such as fibroblasts, endothelial cells, smooth muscle cells and inflammatory cells (stromal cells) [33,34]. Other reports have demonstrated the mechanism by which fibroblasts promote tumor cells growth *in vivo *compared with the *in vitro *model [35,36].

Cell-matrix and cell-cell adhesive interactions play crucial roles in the organization and stability of normal epithelial cells. The conversion of normal cells into malignant cells induces changes in the molecules involved in the adhesion system, which enable a switch to migratory-invasive phenotype in tumor metastasis [37]. These changes are present in ESCC cells, allowing the cells to migrate and invade generating metastasis. The involvement and interaction between fascin and all changes in the membrane are unclear. Malignant tumor cells display a varying degree of resistance to detachment, generating cell-extracellular matrix interactions that are related with the adhesion complex, and this property contributes to tumorigenesis and metastasis [38]. FAK (Focal Adhesine Kynase) is a 125 kDa nonreceptor tyrosine kynase, which regulates cell survival, invasion, migration and cell cycle [39], and its overexpression in human malignant cells has been reported [40-43]. We observed the inhibition of the pFAK by western blot analysis in the KYSE 170 F4 cells. FAK interacts with MAPK cascade regulating cell cycle [44-47]. Furthermore, Integrin α2 is a collagen counter receptor and its knockdown leads to the loss of cell-extracellular matrix adhesion [47]. We observed a considerably low immunoreactivity for integrin α2 through western blot analysis by using the *in vitro *model. The cell-cellular matrix adhesion is required for the continuous activation of the FAK pathway. Therefore, the mechanism of the inhibition of cell growth by fascin knockdown is likely to be partially due to the decrease in the expression of integrin α2. Further research is necessary in order to understand this mechanism.

Cell adhesion and motility functions must be regulated in a coordinated fashion during tumorigenicity. Fascin interacts strongly with β-catenin via an extended region that is known to be necessary for interaction between β-catenin and E-cadherin [22,30,48,49], indicating that fascin and the cytoplasmic domain of E-cadherin compete for the association with β-catenin. From this perspective, it is still unclear which molecular mechanism underlies the role of fascin in the cadherin-catenin complex system in the regulation of cell proliferation, invasion and cell signaling during tumor progression. We found that β-catenin expression was higher in the membrane of KYSE 170F4 cells in both *in vitro *and *in vivo *models, as compared to fascin mock cells, where the expression of β-catenin was located mainly in the cytoplasm and nuclei (Additional files 1 and 2).

## Conclusions

In conclusion, we have identified a potentially important role of fascin in ESCC, where the knockdown of fascin in an ESCC cell line using a siRNA vector induced several effects on cell adhesion, cell growth and the subsequent survival of the ESCC cell line and decreased tumor formation *in vivo*. These findings indicate that fascin may play a role in the prevention of further tumor growth and survival. However, further experiments are necessary in order to determine whether fascin contributes to other pathways involved in transformation, cell growth, survival and metastasis.

## Competing interests

The authors declare that they have no competing interests.

## Authors' contributions

CO carried out protein studies, Tunel analysis, growth assays, immunofluorescence staining, statistical analysis and wrote the manuscript. TI and YH designed the fascin-downregulated cells. AI and ST contributed to the study design and participated in the protein studies, adhesion analysis, growth assays, immunofluorescence staining and statistical analysis. YS and SN drafted the manuscript, collected the samples and participated in its design and coordination. FS contributed in the protein studies, apoptosis analysis and manuscript editing, MM contributed to reviewed specimen pathology and manuscript editing. JG and AP contributed to reviewed manuscript editing and reviewed specimen pathology. All authors read and approved the final manuscript.

## Supplementary Material

Additional file 1 **Immunohistochemical staining for β-catenin**. Photomicrographs of immunohistochemical staining detected β-catenin active pattern in the *in vivo *model tumor using stable transfectant cells with an empty vector (KYSE 170 mock) and with a siRNA vector against fascin (KYSE 170F4). Click here for file

Additional file 2 **Immunofluorescent staining for β-catenin**. Immunofluorescent staining *in vitro*. KYSE 170 mock cells expressing β-catenin in the cytoplasm and nucleus (middle); DAPI (right); Actin. (left). KYSE 170 F4 cells expressing β-catenin in the cytoplasm and membrane (middle); DAPI (right); Actin. (left). Positive control HCT15 cells expressing strongly β-catenin in the nucleus (middle); DAPI (right); Actin. (left). Click here for file
