# Supplementary figures and images for: Effects of small interfering RNAs targeting fascin on human esophageal squamous cell carcinoma cell lines
Source: Diagn Pathol. 2010 Jun 21;5:41. doi: 10.1186/1746-1596-5-41 (PMC2907320; doi:10.1186/1746-1596-5-41)

## Slide 1
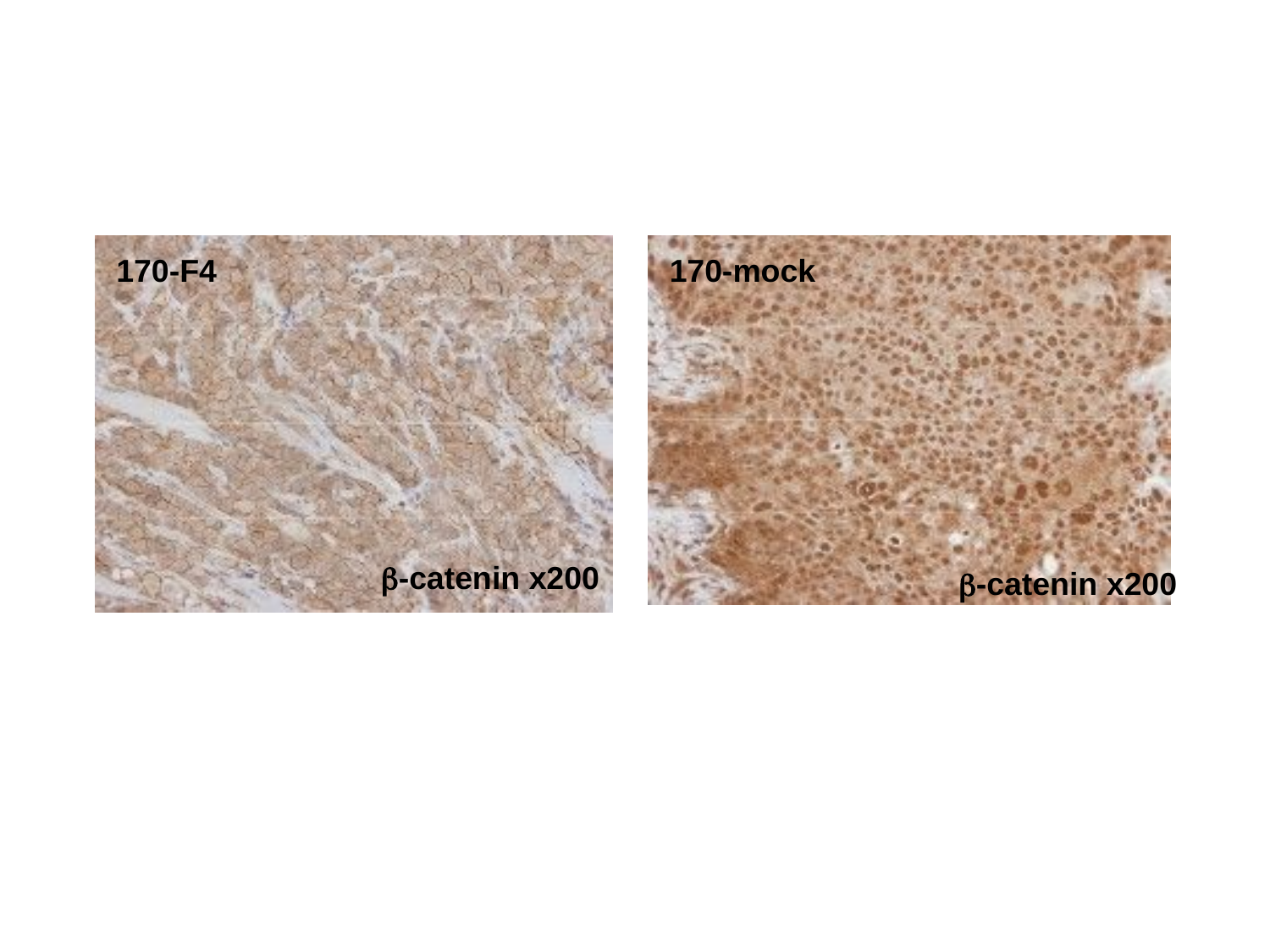

170-F4
170-mock
β-catenin x200
β-catenin x200

Supplement: Additional file 1 — Immunohistochemical staining for β-catenin. Photomicrographs of immunohistochemical staining detected β-catenin active pattern in the in vivo model tumor using stable transfectant cells with an empty vector (KYSE 170 mock) and with a siRNA vector against fascin (KYSE 170F4). [file 1746-1596-5-41-S1.PPT]

## Slide 1
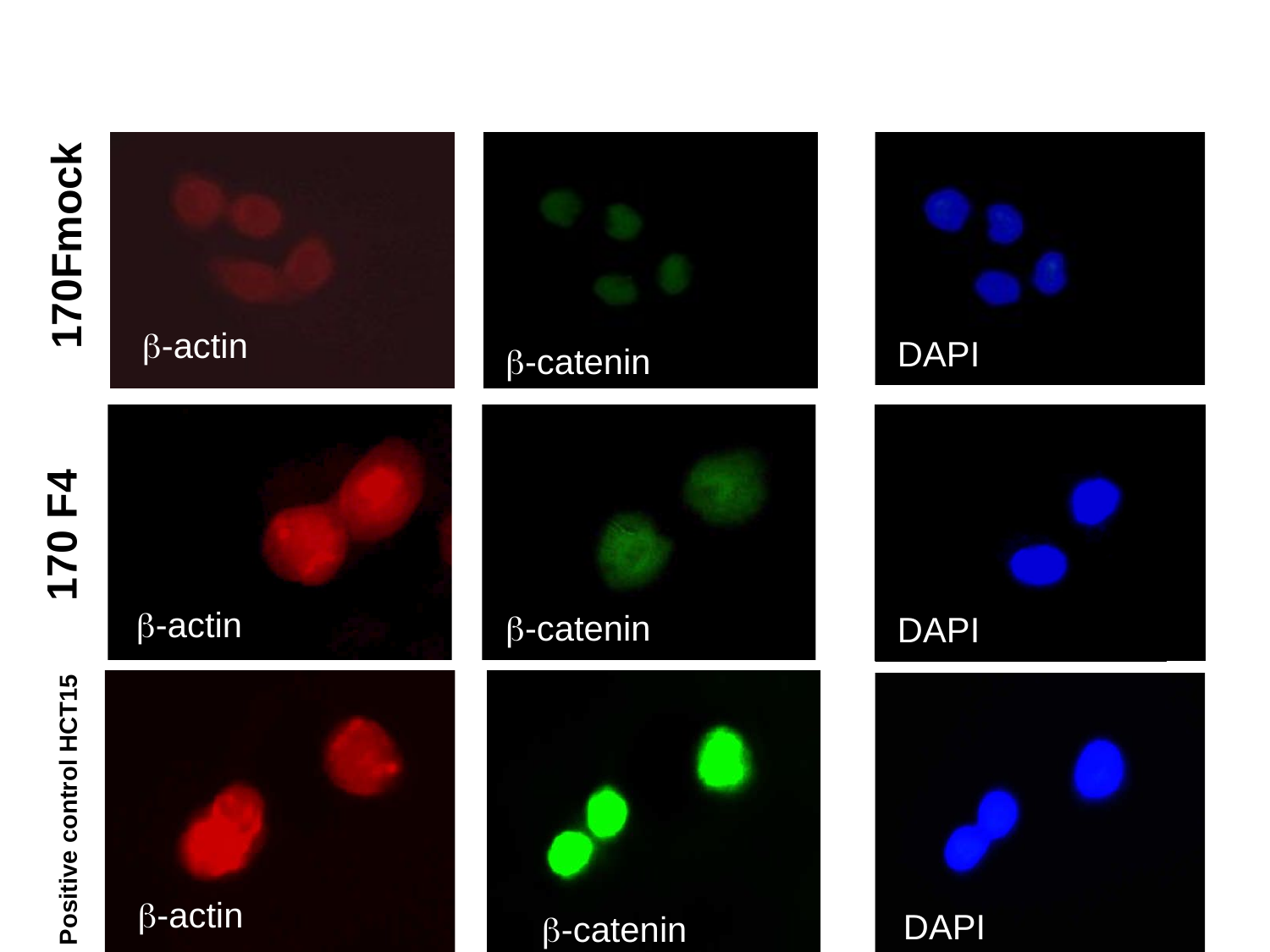

#
170Fmock
β-actin
DAPI
β-catenin
DAPI
170 F4
β-actin
β-catenin
Positive control HCT15
β-actin
DAPI
β-catenin

Supplement: Additional file 2 — Immunofluorescent staining for β-catenin. Immunofluorescent staining in vitro. KYSE 170 mock cells expressing β-catenin in the cytoplasm and nucleus (middle); DAPI (right); Actin. (left). KYSE 170 F4 cells expressing β-catenin in the cytoplasm and membrane (middle); DAPI (right); Actin. (left). Positive control HCT15 cells expressing strongly β-catenin in the nucleus (middle); DAPI (right); Actin. (left). [file 1746-1596-5-41-S2.PPT]
